# Mixed Medullary and Papillary Thyroid Carcinoma in a Patient on Tirzepatide

**DOI:** 10.7759/cureus.107836

**Published:** 2026-04-27

**Authors:** Lauren H Beshay, Jitin Makker, Susan Ahern

**Affiliations:** 1 Endocrinology, Diabetes and Metabolism, David Geffen School of Medicine, University of California Los Angeles, California, USA; 2 Pathology and Laboratory Medicine, David Geffen School of Medicine, University of California Los Angeles, California, USA

**Keywords:** glp1ra, medullary thyroid cancer, papillary thyroid cancer, thyroid nodule, tirzepatide

## Abstract

Medullary thyroid cancer (MTC) stems from thyroid parafollicular C cells and is considered a rare type of neuroendocrine tumor. It can be inherited as part of syndromes, such as familial medullary thyroid cancer (FMTC) and multiple endocrine neoplasia type 2 (MEN 2), or it can arise sporadically. GLP-1 receptor agonist drugs (GLP1 RA), like semaglutide and tirzepatide, carry an FDA black box warning against using them in patients who have a prior history or a family history of MTC or MEN2. This recommendation stems from rodent studies showing thyroid C-cell tumors. Current studies have not confirmed an association between GLP1RAs and risk of differentiated thyroid cancer in human studies. In addition, there is no consensus on screening or evaluating patients for thyroid cancer prior to or during treatment with GLP1 RA. Here, we report a case of mixed medullary and papillary thyroid carcinoma newly diagnosed in a 68-year-old female on tirzepatide for type 2 diabetes who presented with a neck mass.

## Introduction

GLP-1 receptor agonists (GLP-1 RAs) have rapidly influenced the fields of obesity and diabetes management. In preclinical studies on rodents, there were concerns about them inducing C-cell tumors, which led to the US Food and Drug Administration (FDA) warning against their use in patients who have a personal or family history of medullary thyroid carcinoma (MTC) or multiple endocrine neoplasia type 2A or 2B (MEN 2) syndrome. However, the human risk remains uncertain and controversial [1,2].

In rodent studies, GLP-1 RAs stimulate thyroid C-cell hyperplasia and medullary thyroid tumors in a dose- and duration-dependent manner [1,3]. GLP-1 RAs are highly expressed in rodent C-cells, where activation increases calcitonin release and C-cell proliferation via cAMP/PKA and mTOR signaling pathways [4,5]. However, critical species differences exist; humans and non-human primates have substantially lower GLP-1 receptor expression in thyroid C-cells compared to rodents, and GLP-1 RAs do not stimulate calcitonin release in primates [4,6]. The human data linking GLP-1 RAs to MTC have yielded inconsistent results, suggesting more likely cases of incidental correlation rather than causation [3].

MTC is derived from thyroid parafollicular C cells and accounts for 3-4% of all thyroid cancers [1]. Roughly 75% of cases are sporadic, and 25% of cases occur as part of hereditary syndromes associated with germline RET mutations [1]. Recent molecular studies have identified somatic RAS mutations, particularly HRAS, in 10-18% of sporadic RET-negative MTCs, representing an alternative oncogenic pathway [1]. We present a case of sporadic MTC with HRAS mutation in a patient with a prior history of hormone receptor-positive invasive ductal breast carcinoma, who was found to have an incidental thyroid nodule four months after initiation and rapid escalation of doses of a GLP-1 RA.

## Case presentation

A 68-year-old woman with a history of pT1cN0 right breast hormone receptor-positive invasive ductal carcinoma presented for evaluation of a thyroid nodule. She had undergone bilateral mastectomies and completed five years of anastrozole therapy with no evidence of breast cancer recurrence since her surgery nine years prior to this presentation. She also has a history of type 2 diabetes, well-controlled on metformin. She initiated taking tirzepatide four months prior with a monthly dose escalation up to 10 mg subcutaneous weekly injection for weight loss and secondary benefits.

**Figure 1:**
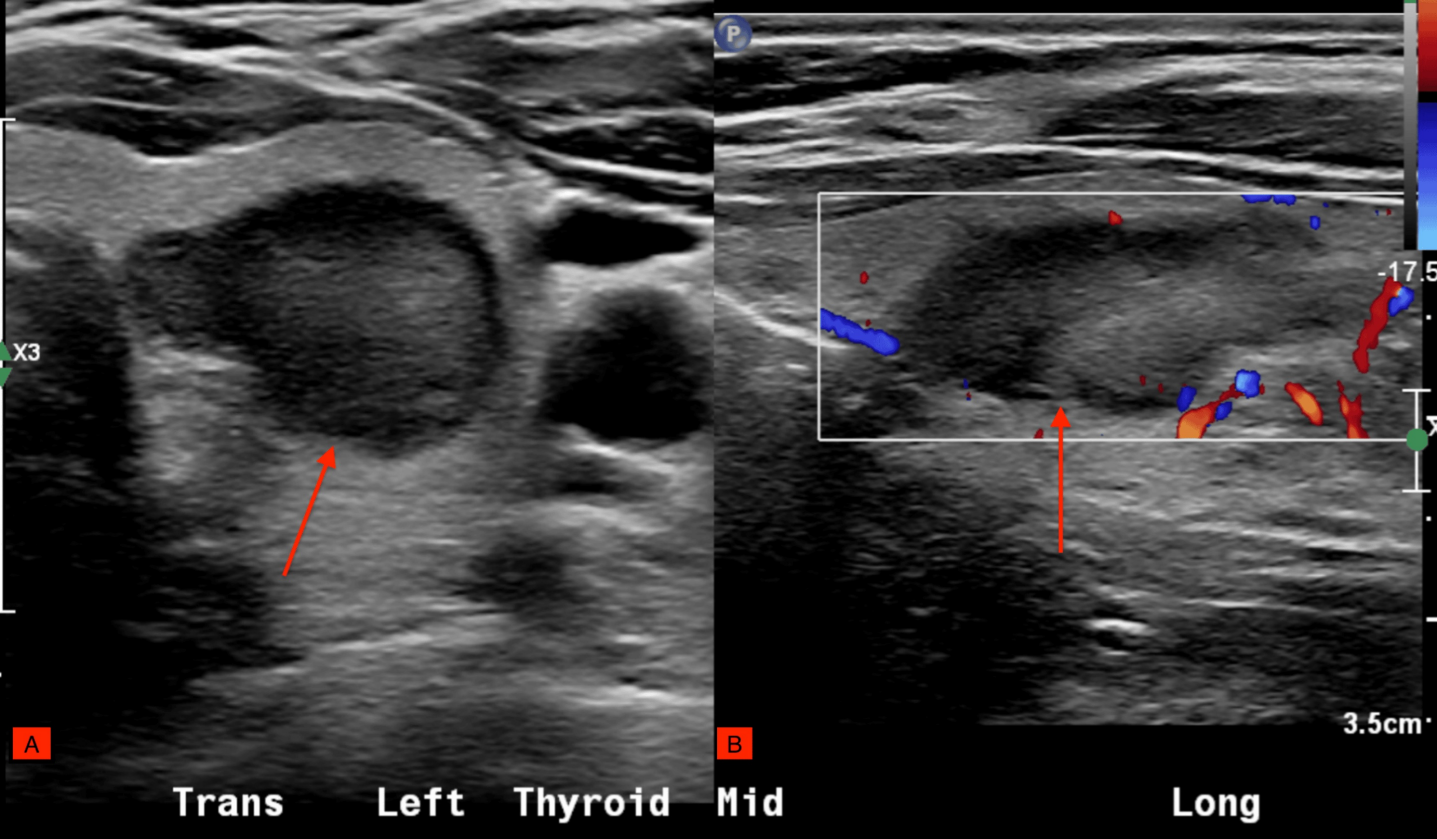
Thyroid ultrasound (24 mm left mid-gland nodule) (A) Trans Mid Left Thyroid. (B) Long Mid Left Thyroid.

**Figure 2:**
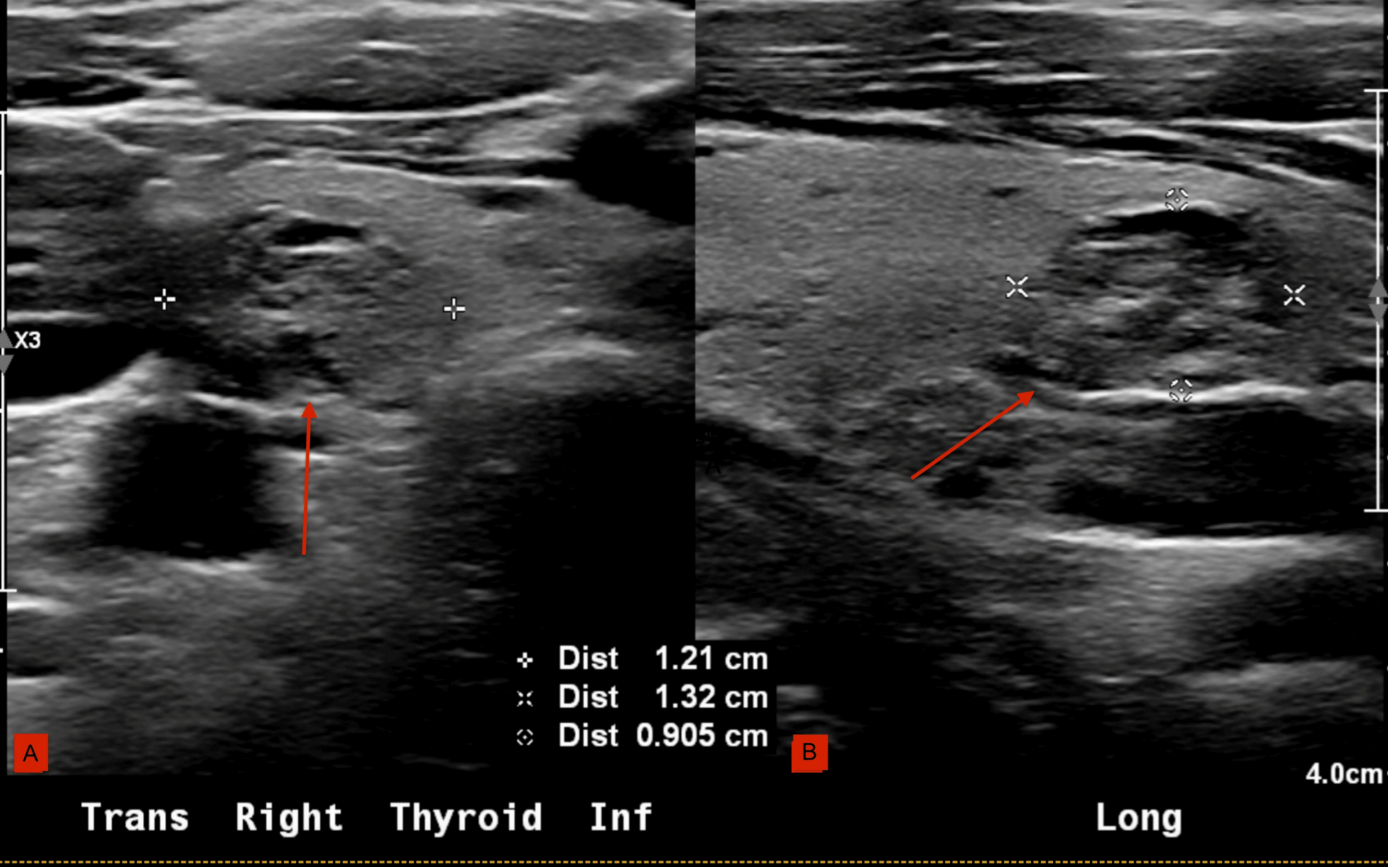
Thyroid ultrasound (13 mm right thyroid nodule) (A) Trans Mid Right Thyroid. (B) Long Mid Right Thyroid.

On routine follow-up, her gynecologist ordered a thyroid biopsy after palpating a left thyroid nodule during routine examination. Thyroid ultrasound with TI-RADS scoring revealed three nodules: a 24 mm left mid-gland nodule, TI-RADS 4 (Figure 1), a 20 mm left inferior gland nodule, TI-RADS 1, and a 13 mm right inferior gland nodule, TI-RADS 2 (Figure 2).

**Figure 3:**
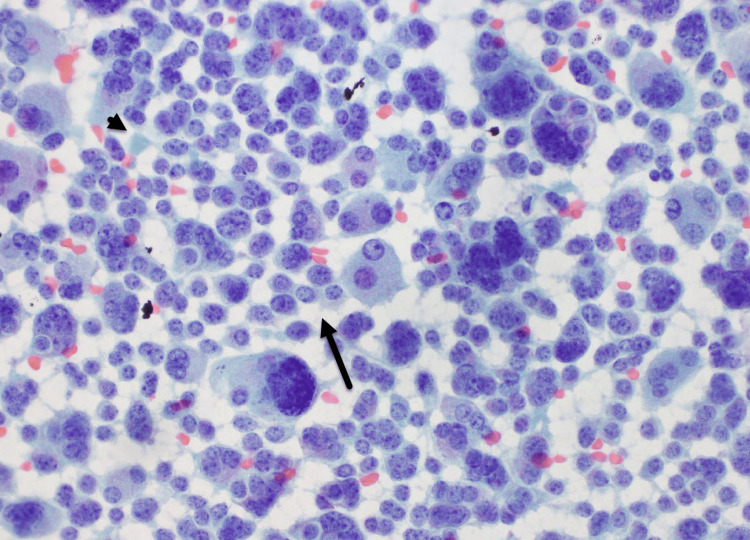
Medullary thyroid cancer fine-needle aspiration pathology Fine-needle aspiration cytology of medullary thyroid carcinoma showing a highly cellular smear composed of dispersed and loosely cohesive plasmacytoid cells with characteristic salt-and-pepper chromatin (arrow). Amorphous extracellular amyloid-like material is present in the background (arrowhead).

Fine needle aspiration of the left mid-gland nodule demonstrated MTC (Positive, HRAS:p.Q61R c.182A>G) (Figure 3). Lung lesions were also discovered simultaneously on a coronary calcium screen ordered by her primary doctor.

At her primary doctor follow-up appointment, she was instructed to stop tirzepatide, and was referred urgently to see Endocrine surgery, Endocrinology, and Interventional Radiology for a lung nodule biopsy. Preoperative laboratory assessment confirmed elevated baseline CEA, and calcitonin (Table 1). Germline genetic testing (Invitae) for RET mutations and MEN2 syndrome was negative, confirming the sporadic nature of the MTC with an HRAS somatic mutation. She reported no family history of thyroid cancer.

**Table 1:** Laboratory values (-) Test not completed post-operatively. CEA: carcinoembryonic antigen.

	Pre-op	Three-month post-op	Reference range
Metanephrine, free	<0.10 nmol/L	-	0.00-0.49 nmol/L
CEA	20.1 ng/ml	1.2 ng/ml	<3.1 ng/ml
Calcitonin	207 pg/ml	2 pg/ml	0.0-5.1 pg/ml
Throglobulin		<0.2 ng/ml	3-40 ng/ml

**Figure 4:**
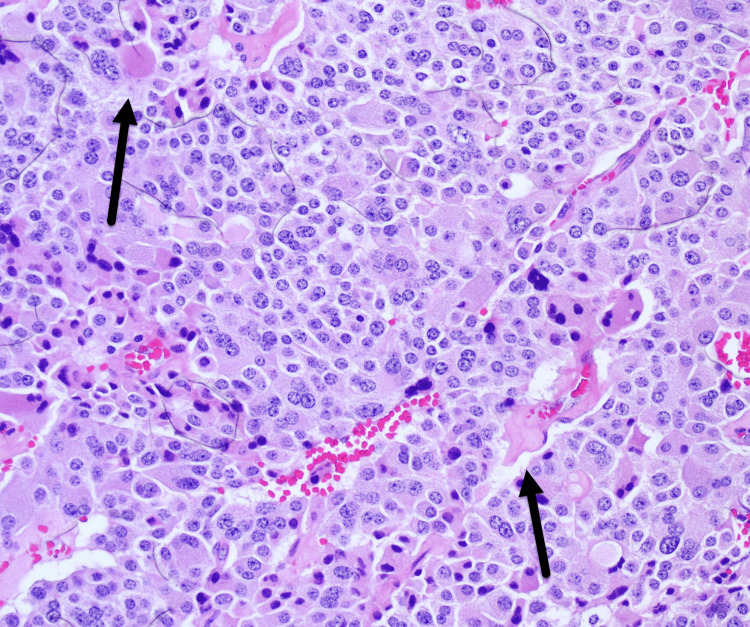
Resection H&E Medullary thyroid cancer Medullary thyroid carcinoma demonstrating nests and trabeculae of tumor cells with plasmacytoid to spindle morphology and neuroendocrine-type (‘salt-and-pepper’) chromatin. Amorphous eosinophilic extracellular material is present (arrow).

**Figure 5:**
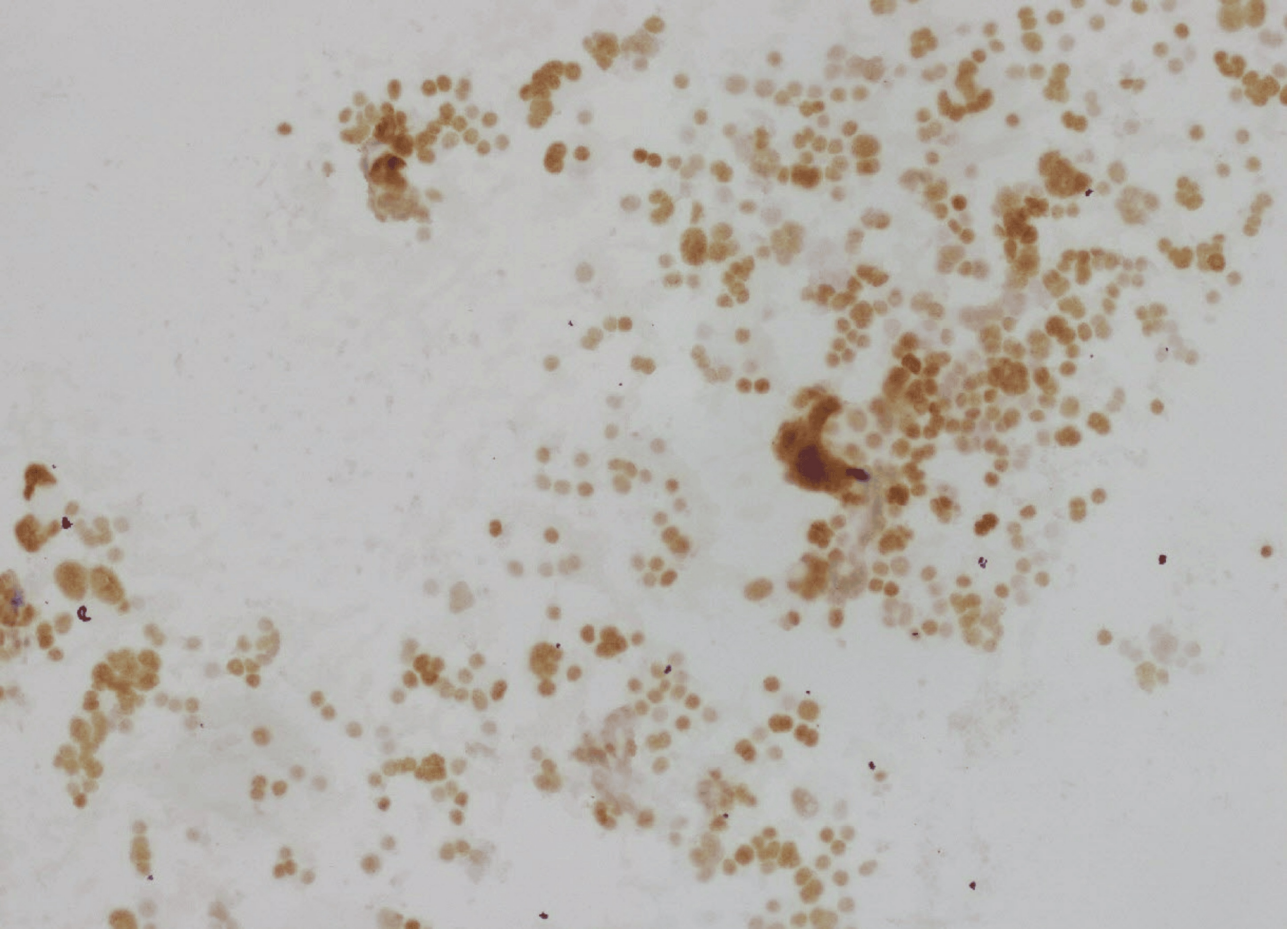
Immunohistochemistry stain of medullary thyroid cancer Immunohistochemistry stain (INSM1) - medullary carcinoma is showing nuclear positivity.

**Figure 6:**
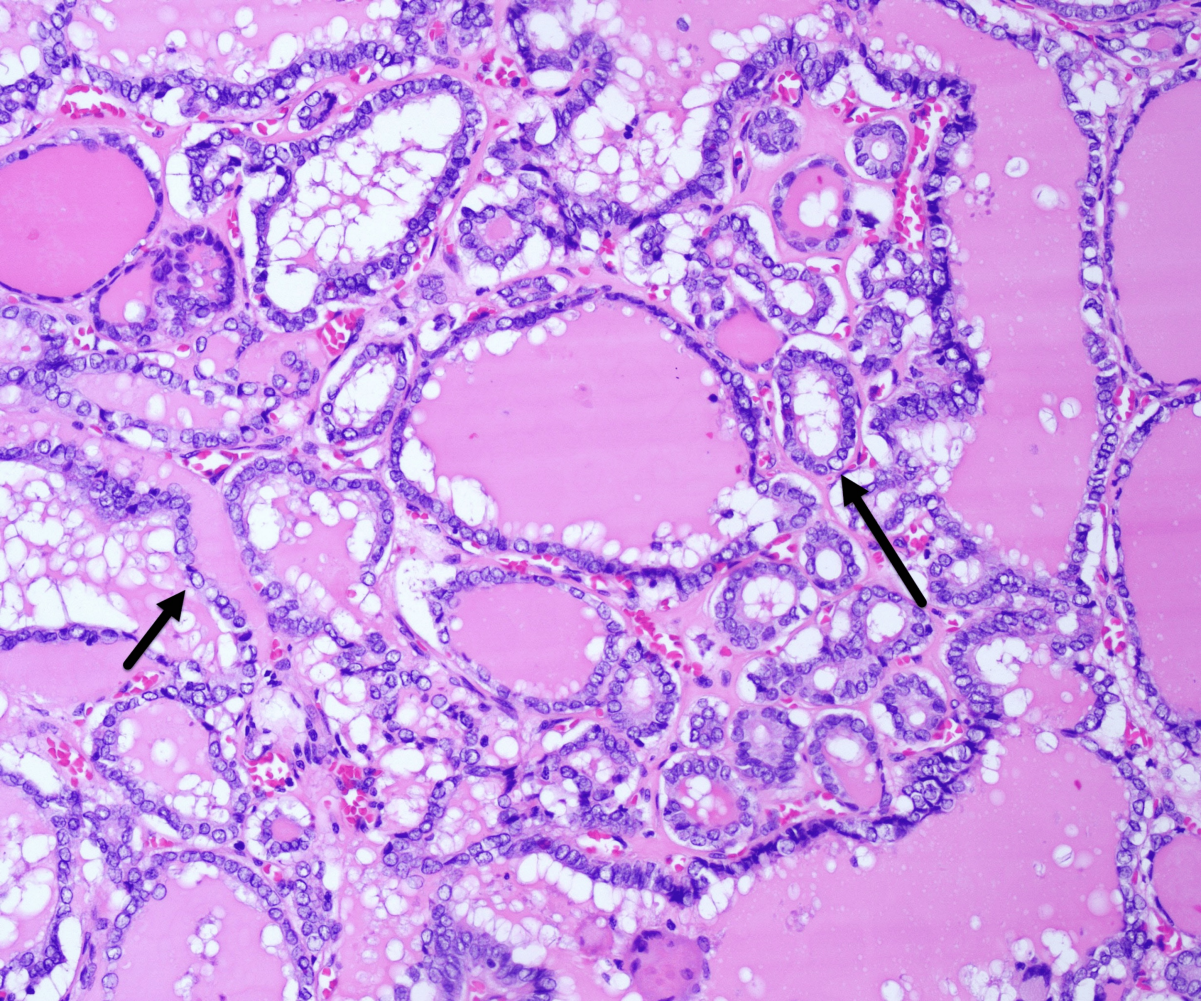
Papillary thyroid cancer from surgical pathology Incidental papillary thyroid carcinoma showing characteristic nuclear features (arrow), including nuclear clearing, grooves, and rare intranuclear pseudoinclusions.

Intraoperatively, a left thyroid tumor with minor anterior extrathyroidal extension was noted. She underwent a total thyroidectomy with central neck dissection. Final pathology revealed a primary tumor of 2.0 cm MTC with lymphovascular invasion, no extrathyroidal extension, 0/6 lymph nodes positive for metastasis (staging: pT1bN0a) (Figures 4 and 5). Incidental findings included two foci of papillary thyroid carcinoma, well-demarcated follicular subtype (0.2 and 0.3 cm) on the right (Figure 6).

Three months post-operatively, tumor marker surveillance demonstrated excellent biochemical response to surgery (Table 1). A lung nodule biopsy was also performed and was negative for malignancy. The patient was counseled further on the risk of GLP1RA use with the new diagnosis of MTC.

## Discussion

Based on rodent studies, GLP1 RAs should be avoided in patients who have a personal or family history of MTC. However, the human data yield inconsistent results. A large Scandinavian cohort study found no increased thyroid cancer risk with GLP-1 RAs versus DPP-4 inhibitors (HR 0.93; 95% CI: 0.66-1.31) over a mean 3.9-year follow-up [7]. Similarly, a meta-analysis of clinical trials from Novo Nordisk (Bagsværd, Denmark) showed no association (HR 0.87; 95% CI: 0.58-1.29) [8].

Conversely, a French case-control study reported increased risk of all types of thyroid cancers (adjusted HR 1.58; 95% CI: 1.27-1.95) and MTC specifically (adjusted HR 1.78; 95% CI: 1.04-3.05) within 1-3 years of GLP-1 RA use [9]. A meta-analysis of randomized trials showed a modest increase in overall thyroid cancer risk (OR 1.52; 95% CI: 1.01-2.29), though with a fragility index of only 1 [10].

Recent evidence suggests that the observed association may reflect enhanced surveillance rather than causation. A large US cohort study demonstrated that thyroid cancer risk was elevated only in the first year after GLP-1 RA initiation, with no sustained increase thereafter [3]. This temporal specificity argues against true carcinogenesis, as the typical latency period for thyroid cancer development (approximately 2.5 years from known carcinogens) would not support cancer emergence within months of drug exposure [3]. The pattern is more consistent with the detection of pre-existing thyroid cancers through heightened clinical awareness and surveillance [3].

Reported human cases of MTC in GLP-1 RA users also typically represent pre-existing disease rather than drug-induced carcinogenesis. For example, a case involved a man in his 30s with obesity and hypothyroidism who underwent pre-treatment screening before starting a GLP-1 RA for weight loss. His calcitonin level returned elevated at 131 pg/mL (normal <10 pg/mL) [11]. Thyroid ultrasound revealed bilateral subcentimeter nodules, and total thyroidectomy showed bilateral 0.8 cm MTC [11]. Genetic testing revealed a germline RET mutation, confirming MEN 2A syndrome [11]. This case represents the incidental detection of pre-existing familial MTC during appropriate pre-treatment screening.

Our case also illustrates another likely incidental sporadic MTC with mixed PTC. Her prior known history of breast cancer prompted a more proactive role in detecting and referring for the biopsy of her thyroid nodule. The patient had initiated a GLP1 RA only four months prior to diagnosing her thyroid cancer, which would make the temporal causation unlikely despite rapid escalation in doses in this short period. Given also the incidental PTC on final pathology, this would have a different pathogenesis than the C-cell hyperplasia postulated from GLP1 RA use in rodent studies. Lastly, the co-occurrence of breast cancer and thyroid cancer in this patient is likely more noteworthy than the GLP1 RA use. Until further data arise on GLP1RA use and risk of differentiated thyroid cancer in human studies, moving forward this patient was advised against future use of any GLP1 RA for treatment of her type 2 diabetes or weight management.

## Conclusions

For the general population, current evidence does not support routine thyroid screening before initiating or during management with GLP-1 RA therapy. The metabolic and cardiovascular benefits of GLP-1 RAs outweigh the uncertain thyroid cancer risk in most patients, particularly given the low absolute risk. Based on the current data, there is a lack of evidence that GLP1 RA causes MTC in humans. This case demonstrates a likely incidental rather than causative link between GLP1 RA use and the diagnosis of PTC and MTC. Whether we will continue to avoid the use of GLP1RAs in patients with personal or family history of MTC or MEN2 based on rodent data is still unclear.
